# Remote programming versus standard in-person programming following deep brain stimulation in patients with Parkinson’s disease: a randomised controlled trial

**DOI:** 10.1016/j.eclinm.2026.104070

**Published:** 2026-07-09

**Authors:** Xiaonan Wan, Yaodong Zhou, Peng Huang, Yixin Pan, Zhengyu Lin, Zhitong Zeng, Yuyan Tan, Chencheng Zhang, Paul Shotbolt, Dianyou Li

**Affiliations:** a Department of Neurosurgery, Center for Functional Neurosurgery, Ruijin Hospital, Affiliated with Shanghai Jiao Tong University School of Medicine, Shanghai, China; b Institute of Psychiatry, Psychology and Neuroscience, King’s College London, London, UK; c Department of Neurology & Institute of Neurology, Ruijin Hospital, Affiliated with Shanghai Jiao Tong University School of Medicine, Shanghai, China; d Clinical Neuroscience Center, Ruijin Hospital, Shanghai, China

**Keywords:** Deep brain stimulation, Motor symptoms, Parkinson disease, Remote programming, Telemedicine

## Abstract

**Background:**

Remote programming (RP) offers a convenient alternative for patients with Parkinson’s disease (PD) who have undergone deep brain stimulation (DBS). However, its clinical efficacy remains underexplored. We aimed to compare the treatment effects of RP and in-person standard programming (SP) in this population.

**Methods:**

We conducted an open-label, non-inferiority randomised controlled trial at a single centre in China between October 2023 and February 2025. This study included PD patients who underwent bilateral subthalamic nucleus DBS surgery. Participants were randomly assigned (1:1) to either the RP or SP group. The primary outcome was the improvement rate in motor symptoms from baseline to 6-month follow-up, assessed using the Unified Parkinson’s Disease Rating Scale (UPDRS). Secondary outcomes included changes in non-motor symptoms, quality of life, and patients’ subjective experiences of the programming sessions. This study is registered at ClinicalTrials.gov (NCT06078397).

**Findings:**

A total of 50 patients were enrolled; 20 in the SP group and 21 in the RP group completed the 6-month follow-up. With a non-inferiority margin of 10%, the improvement rate in the total UPDRS-III score in the RP group was found to be non-inferior to that in the SP group, as the one-sided lower bound of the 95% confidence interval (−0.033) exceeded −0.100 (−10%). No significant differences were observed between the two groups in non-motor symptoms, quality of life, or subjective programming experiences (all p > 0.05).

**Interpretation:**

RP offers short-term motor improvements that are non-inferior to in-person SP in PD patients following DBS surgery, which also yields comparable outcomes in non-motor symptoms and quality of life.

**Funding:**

Dianyou Li was funded by 10.13039/100017950Shanghai Municipal Health Commission (Grant No. 202140181), Shanghai Science and Technology Commission (Grant No. 22Y11903900), Guangci Innovative Technology Launch Program (Grant No. GCQH202205), and the 10.13039/501100001809National Natural Science Foundation of China (82571678 to D.L.); Xiaonan Wan was funded by King’s College London-China Scholarship Council Scholarship.

**Research in context**

**Evidence before this study**

We searched PubMed for articles published from database inception to January 1, 2025, using combinations of the terms “Parkinson’s disease”, “deep brain stimulation”, “remote programming”, and “telemedicine”. Previous studies have demonstrated the feasibility, safety, and patient satisfaction associated with remote programming after deep brain stimulation (DBS). However, most available evidence has been derived from retrospective studies, single-arm cohorts, or real-world experiences.

**Added value of this study**

To our knowledge, this is the first randomized non-inferiority trial evaluating remote programming in patients with Parkinson’s disease after DBS surgery. We found that remote programming achieved motor improvements that were non-inferior to those obtained with standard programming during the first six months after surgery. The two groups showed comparable changes in motor and non-motor symptoms, quality of life, and medication use, with similar rates of adverse events. Patients receiving remote programming also reported high levels of satisfaction, supporting the feasibility and acceptability of this approach.

**Implications of all the available evidence**

Taken together with previous observational studies, our findings support remote programming as an effective and practical alternative to conventional in-person programming for patients with Parkinson’s disease after DBS surgery. Remote programming may improve access to specialized care and reduce the burden associated with repeated hospital visits, particularly for patients living far from DBS centers. Further multicenter studies with longer follow-up are warranted to evaluate the long-term efficacy, safety, and cost-effectiveness of this approach.

## Introduction

Deep brain stimulation (DBS) is a cost-effective, invasive treatment for patients with Parkinson’s disease (PD), modulating neural activity through controlled electrical currents generated by an implantable pulse generator (IPG).^1^^,^^2^ Following DBS surgery, the IPG must be adjusted through standard programming (SP) sessions to achieve optimal therapeutic outcomes.^3^ However, the need for frequent long-distance travel to specialized medical centers poses substantial financial and logistical burdens, particularly for patients living far from DBS centers.^4^^,^^5^

As an alternative to SP, internet-based remote programming (RP) is a novel technology that enables physicians to conduct programming sessions for patients with DBS remotely. In China, RP became operational in 2015 and expanded rapidly during the COVID-19 pandemic. Currently, over 10,000 sessions are conducted annually.^6^^,^^7^

Previous studies have explored the necessity, safety, and efficacy of RP.^8^, ^9^, ^10^ Our recent study demonstrated that RP significantly reduced the cost of DBS programming in China.^5^ However, its treatment effect on PD patients following DBS surgery remains to be fully established. Xu et al. conducted a follow-up study of 32 PD patients who underwent RP, using video-based assessments, without evaluating symptoms related to rigidity or balance function.^11^ Studies by Nie et al. and Chen et al. retrospectively compared non-motor symptoms and programming burden between patients receiving RP or SP, but motor symptom was not assessed in either study.^12^^,^^13^

To address these gaps, we conducted a single center randomized controlled trial to compare the effects of RP and SP in PD patients during the six months following DBS surgery, providing real-world follow-up evidence for the efficacy of RP in this population.

## Methods

### Study design and participants

This study was designed as a single-center, randomized, open-label, non-inferiority clinical trial, which was approved by the Institutional Review Board of Ruijin hospital (Clinical Ethics Review (2023) No. 231) and registered at ClinicalTrials.gov (NCT06078397). Patient recruitment for this study began on October 19, 2023, and concluded on August 25, 2024, with follow-up completed by February 18, 2025. Written informed consent was obtained from all participants prior to enrollment.

Eligible participants were individuals aged 18–75 years who met the following criteria: (1) a primary diagnosis of PD; (2) had undergone bilateral subthalamic nucleus (STN)-DBS surgery; (3) had accurate lead placement confirmed by postoperative CT co-registered with preoperative MRI; (4) were implanted with a DBS system that supports RP function (PINS: G106R IPG with L302/L301 electrodes; SceneRay: SR1101 IPG with 1200/1210 electrodes); (5) had access to a stable Internet connection, and were capable of participating in RP; (6) were able to communicate fluently through RP system after instruction; and (7) understood the potential risks and benefits of participation, agreed to the study procedures and follow-up requirements, and provided informed consent to comply with the study protocol.

Exclusion criteria were as follows: (1) preoperative cognitive assessment indicating moderate to severe cognitive impairment; (2) serious postoperative complications such as stroke, encephalitis, or wound infection; (3) lack of cooperation, inability to comprehend the study protocol, or inability to provide informed consent for any reason; (4) inability to ensure a stable internet connection or to provide a 4 × 1.5 m space required for RP sessions; and (5) any other conditions deemed unsuitable for participation by the researchers.

Baseline characteristics recorded at the time of the surgical decision included age, gender, cognitive function, PD status, and neuropsychiatric symptoms. Patients were classified into three PD subtypes—tremor-dominant (TD) and postural instability/gait disorder (PIGD)—based on scores from the Unified Parkinson’s Disease Rating Scale (UPDRS).^14^ Preoperative cognitive function was evaluated using the Mini-Mental State Examination (MMSE).^15^

### Randomization and blinding

Block randomization with a fixed block size of 2 was performed using a computer-generated random number sequence to evenly assign participants in a 1:1 ratio to either the RP group (experimental) or the SP group (active comparator), ensuring balanced group sizes. The randomization was not blinded to participants or researchers. Based on the randomization results, each patient received their initial and subsequent programming session (via SP or RP) one month after DBS surgery.

### Procedures

The general RP process—including appointment scheduling, device connectivity, parameter adjustment, and effect evaluation—has been described in a previous publication.^9^ During the initial programming session, the physician completed the ‘DBS Initial Programming Record Form’ (Supplementary Table S1). This form, developed by the research team, was designed to systematically document the minimum stimulation intensity required to induce side effects (e.g., dizziness, limb numbness, diplopia) and to achieve significant symptom improvement (e.g., tremor, bradykinesia) for each electrode contact. These data were used to determine the therapeutic window of each contact, serving as a guide for subsequent sessions.

In addition, both groups underwent a scheduled postoperative programming session at three months after surgery. Patients were permitted to request additional programming sessions based on individual needs, reflecting real-world clinical practice. All postoperative programming was performed by the same experienced physician (D.L.) to minimize inter-physician variability and ensure consistency in treatment delivery and outcome assessment.

Both patients and clinicians accessed the RP system using secure usernames and passwords. Data generated during each session were stored in a secure, cloud-based database, ensuring the privacy and security of patient information.^16^^,^^17^

### Outcome measures

A set of scales was used to assess multiple health indicators relevant to PD. The primary outcome of this study was the improvement of motor symptoms from baseline to 6-month follow-up, assessed by the UPDRS-III scale.^18^ The cost-effectiveness was also analyzed and will be reported separately.

Secondary outcomes included improvement in other PD symptoms, assessed using the UPDRS Parts I, II, and IV^18^; enhancement of quality of life, measured by the 8-item Parkinson’s Disease Questionnaire (PDQ-8)^19^ and the EQ-5D Visual Analogue Scale (EQ-VAS)^20^; changes in PD medication dosage, expressed as the levodopa equivalent daily dose (LEDD, in milligrams); and improvement in neuropsychiatric symptoms, specifically depression and anxiety, evaluated using the Beck Depression Inventory (BDI)^21^ and the Beck Anxiety Inventory (BAI),^22^ respectively.

All baseline (within one week prior to DBS surgery) and follow-up (six months after surgery) assessments were performed in person at our DBS center. During each assessment, patients were in a medication washout state, having discontinued antiparkinsonian drugs for at least 12 h (overnight medication withdrawal). The postoperative UPDRS-III motor assessment was performed with DBS stimulation. Motor symptoms were video-recorded and independently scored by two MDS-certified raters who were blinded to group allocation. All other data were collected while DBS stimulation was ON, using structured interviews and self-administered questionnaires.

In addition, patients were asked to complete the Patient Global Impression of Change (PGIC) scale via an online questionnaire within 72 h after each programming session. The PGIC is a 7-point scale that captures the patient’s subjective assessment of overall improvement. Patients rated their perceived change as follows: 1– very much worse, 2– much worse, 3– a little worse, 4– no change, 5– a little improved, 6– much improved, and 7– very much improved.

### Statistical analysis

All variables in this study were presented as frequency (percentage) for categorical variables or mean ± standard deviation for continuous variables. Group differences in categorical variables were analyzed using the Mantel-Haenszel chi-square test. For continuous variables, normality was assessed using the Shapiro–Wilk test and Q–Q plots. A two-tailed p-value of less than 0.05 was considered statistically significant.

For variables that followed a normal distribution, paired t-tests were used to compare symptom improvements (e.g., UPDRS, PDQ-8 scores) between baseline and follow-up within each group, while independent samples t-tests were used to compare improvements between the RP and the SP group. For variables that did not follow a normal distribution, the Wilcoxon signed-rank test was used for within-group comparisons, and the Mann–Whitney U test was used for between-group comparisons. To compare the overall programming experience between the two groups, mean PGIC scores were calculated on a per-patient basis. As the analyses of secondary outcomes in this study were exploratory and aimed at identifying potential trends, adjustments for multiple comparisons were not performed.

The primary outcome was the percentage improvement in UPDRS-III score from baseline to the 6-month follow-up. Non-inferiority t-test was conducted to compare the percentage improvement between the two groups. The established six-month effect of STN-DBS, corresponding to an improvement of 47.68% (95% CI, 42.05–53.31), was used as historical evidence.^23^ To provide a conservative estimate of the active control effect, the lower limit of the confidence interval (42.05%) was adopted as M1. A non-inferiority margin of 10 percentage points was prespecified as the maximum clinically acceptable loss of efficacy compared with SP. This margin preserves approximately 76% of the historical treatment effect, as it allows a maximum loss of 10 percentage points from M1 (i.e., [42.05−10]/42.05 ≈ 76%).^24^

Given these parameters—with a one-side significance level of 5%, a power of 80%, and a 1:1 group allocation ratio—and accounting for an anticipated dropout rate of up to 20%, the required sample size was calculated to be 50 participants based on the following formula^25^:

$$n_{A}=\kappa n_{B}\;and\;n_{B}=\left(1+\frac{1}{\kappa }\right){\left(\sigma \frac{z_{1-\alpha }+z_{1-\beta }}{\mu_{A}-\mu_{B}-\delta }\right)}^{2}$$

$$1-\beta =\Phi \left(z-z_{1-\alpha }\right)+\Phi \left(-z-z_{1-\alpha }\right),z=\frac{\mu_{A}-\mu_{B}-\delta }{\sigma \sqrt{\frac{1}{n_{A}}+\frac{1}{n_{B}}}}$$

κ represents the sample size ratio between the two groups; σ denotes the standard deviation; Φ is the cumulative distribution function of the standard normal distribution; Φ^−1^ is the inverse (quantile) function of the standard normal distribution; α is the Type I error probability; β is the Type II error probability; and δ represents the non-inferiority margin.

The primary efficacy analysis was performed in the per-protocol population, which included participants who adhered to the allocated intervention and completed the 6-month follow-up assessment. To assess the robustness of the findings and the potential impact of missing data, sensitivity analyses were conducted according to the intention-to-treat principle, including all randomized participants. Missing outcomes were first explored using best–worst and worst–best case scenarios. In the best–worst scenario, participants with missing outcomes in the RP group were assigned the most favorable observed outcome, whereas those in the SP group were assigned the least favorable observed outcome; the reverse assumptions were applied in the worst–best scenario. Missing values were further addressed using multiple imputation with a monotone regression method. The imputation model included treatment group, sex, age, and baseline outcome score.

Data analysis was performed using SAS software (version 9.4; SAS Institute, Cary, NC, USA), and data management was conducted using Microsoft Excel (version 16.95; Microsoft Corporation, Redmond, WA, USA).

### Role of the funding source

The funder of the study had no role in study design, data collection, data analysis, data interpretation, or writing of the report.

## Results

**Fig. 1:**
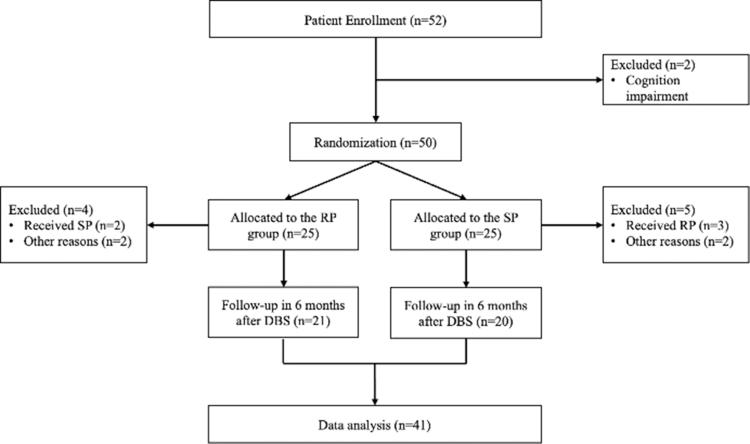
Participant flow diagram.

This study included 50 patients with PD who had undergone DBS surgery. The patients were evenly assigned to the SP group and the RP group. Of these, 20 patients in the SP group and 21 patients in the RP group completed the 6-month postoperative follow-up (Fig. 1).

In the SP group, five patients were excluded: three switched to RP due to travel restrictions, and two declined further participation for personal reasons. In the RP group, four patients were excluded: two declined RP due to concerns about its efficacy, and two exited this study for personal reasons.

Baseline characteristics of the two groups are presented in (Table 1). The SP group consisted of 10 females and 19 TD patients, while the RP group included 13 females and 21 TD patients, no patients met the criteria for the indeterminate subtype. The groups differed in age (mean ± SD: 60.56 ± 10.42 versus 61.76 ± 9.68 years), disease duration (12.36 ± 5.89 versus 13.36 ± 12.08 years), distance from home to the medical center (468.72 ± 386.46 km versus 479.12 ± 375.46 km), preoperative cognitive function (MMSE: 22.92 ± 3.00 versus 24.00 ± 3.20), levodopa equivalent daily dose (LEDD: 811.97 ± 323.11 mg versus 787.27 ± 342.56 mg), and levodopa response (54.67 ± 11.27% versus 55.90 ± 10.95%). None of these differences were statistically significant (p > 0.05).

**Table 1:** Baseline characteristics of participants.

Item	Standard programming group	Remote programming group	p value^a^
Sample size	N = 25	N = 25	
Sex (female)- n (%)	10 (40%)	13 (52%)	0.399
Age (year)	60.56 ± 10.42	61.76 ± 9.68	0.690
Disease duration (Year)	12.36 ± 5.89	13.36 ± 12.08	0.565
Distance from residence to DBS center (km)	468.72 ± 386.46	479.12 ± 375.46	0.793
MMSE score	22.92 ± 3.00	24.00 ± 3.20	0.231
LEDD	811.97 ± 323.11	787.27 ± 342.56	0.794
LR (%)	54.67 ± 11.27	55.90 ± 10.95	0.626
Subtype- n (%)			0.484
TD	19 (76%)	21 (84%)	
PIGD	6 (24%)	4 (16%)	

MMSE = Mini-Mental State Examination; LEDD = Levodopa Equivalent Daily Dose; LR = Levodopa Response; TD = Tremor-Dominant; PIGD = Postural Instability/Gait Disorder.

Data was shown as mean ± SD or count (proportion).

a Between-group comparisons were performed using independent-samples t tests or Mann–Whitney U tests, depending on data distribution.

The changes in clinical symptoms from baseline to the follow-up period in both groups are presented in (Supplementary Table S2). Compared to the preoperative baseline, both groups showed significant improvements in motor symptoms, non-motor symptoms, quality of life, and PD medication six months after DBS surgery (p < 0.05). With a non-inferiority margin (|δ_L_|) of 10%, the improvement rate in the total UPDRS-III score in the RP group was non-inferior to that in the SP group, as the one-sided lower bound of the 95% confidence interval (−0.033) exceeded −0.100 (−10%), and sensitivity analyses further supported the non-inferiority under most conditions (Table 2).

**Table 2:** Non-inferiority test and sensitivity analyses of improvement rate of motor symptoms between two groups.

	Remote programming group	Standard programming group	LB_95_ (one-sided CI)^a^
Per-protocol analyses	37.80 ± 14.17	35.93 ± 10.63	−0.033^b^
Sensitivity analyses			
Worse-best case	34.48 ± 15.08	40.43 ± 13.19	−0.112
Best-worst case	41.83 ± 16.01	32.07 ± 12.30	0.045^b^
Multiple imputation 1	–	–	−0.054^b^
Multiple imputation 2	–	–	−0.052^b^

Data was shown as mean ± SD.

a LB95 (one-sided CI) refers to the lower bound of the one-sided 95% confidence interval for the mean difference between the two groups.

b Non-inferiority is established if LB_95_ (one-sided CI) > −10% (the predefined non-inferiority margin).

A comparison of symptom improvement between the SP and RP groups is presented in (Table 3). No statistically significant differences were observed in the changes of motor or non-motor symptoms between the two groups: improvements in bradykinesia (7.71 ± 7.89 versus 7.60 ± 3.62), rigidity (4.57 ± 3.30 versus 4.30 ± 3.57), tremor (6.30 ± 5.88 versus 7.52 ± 5.35), and axial symptoms (3.85 ± 2.46 versus 5.10 ± 3.53) were comparable. Similarly, there were no significant differences in the improvement of non-motor symptoms (UPDRS-I: 3.30 ± 5.45 versus 2.14 ± 3.55), activities of daily living (UPDRS-II: 3.65 ± 5.98 versus 3.81 ± 6.55), motor complications (UPDRS-IV: 2.30 ± 2.75 versus 1.33 ± 1.85), PD medication usage (LEDD: 468.07 ± 317.63 mg versus 398.21 ± 242.53 mg), depressive symptoms (BDI: 4.55 ± 6.47 versus 4.14 ± 7.41), anxiety symptoms (BAI: 5.60 ± 7.85 versus 3.90 ± 7.26), and quality of life (PDQ-8: 3.00 ± 4.30 versus 3.33 ± 4.43; EQ-VAS: −14.50 ± 18.70 versus −9.43 ± 16.54), with all p-values > 0.05.

**Table 3:** Exploratory analyses of symptom improvement in two groups.

	Standard programming group		Remote programming group		p value^b^
	Baseline	Change^a^	Baseline	Change^a^	
Sample size	N = 20		N = 21		
UPDRS-III					
Total	61.05 ± 11.12	22.05 ± 7.88	63.00 ± 18.08	24.90 ± 14.85	–
Bradykinesia	28.25 ± 4.58	7.60 ± 3.62	28.14 ± 7.88	7.71 ± 7.89	0.953
Rigidity	12.75 ± 3.32	4.30 ± 3.57	12.14 ± 3.15	4.57 ± 3.30	0.844
Tremor	10.40 ± 7.00	6.30 ± 5.88	12.29 ± 6.30	7.52 ± 5.35	0.521
Axial symptoms	9.65 ± 3.31	3.85 ± 2.46	10.43 ± 4.28	5.10 ± 3.53	0.200
LEDD	855.57 ± 340.86	468.07 ± 317.63	825.59 ± 357.86	398.21 ± 242.53	0.432
BDI	10.10 ± 8.13	4.55 ± 6.47	9.10 ± 7.62	4.14 ± 7.41	0.626
BAI	8.95 ± 8.68	5.60 ± 7.85	8.24 ± 7.73	3.90 ± 7.26	0.477
UPDRS-I	9.75 ± 6.29	3.30 ± 5.45	8.10 ± 5.55	2.14 ± 3.55	0.409
UPDRS-II	17.95 ± 8.15	3.65 ± 5.98	16.05 ± 6.61	3.81 ± 6.55	0.936
UPDRS-IV	7.40 ± 2.56	2.30 ± 2.75	6.29 ± 2.49	1.33 ± 1.85	0.193
PDQ-8	8.15 ± 3.86	3.00 ± 4.30	7.05 ± 4.95	3.33 ± 4.43	0.674
EQ-VAS	59.70 ± 18.62	−14.50 ± 18.70	65.19 ± 16.19	−9.43 ± 16.54	0.363

LEDD = Levodopa Equivalent Daily Dose; BDI = Beck Depression Inventory; BAI = Beck Anxiety Inventory; UPDRS = Unified Parkinson’s Disease Rating Scale; PDQ-8 = 8-item Parkinson’s Disease Questionnaire; EQ-VAS = EQ-5D Visual Analogue Scale.

Data was shown as mean ± SD.

a Change = Scores at baseline–scores at follow-up.

b Between-group comparisons were performed using independent-samples t tests or Mann–Whitney U tests, depending on data distribution. As the analyses of secondary outcomes in this study were exploratory and aimed at identifying potential trends, adjustments for multiple comparisons were not performed.

**Fig. 2:**
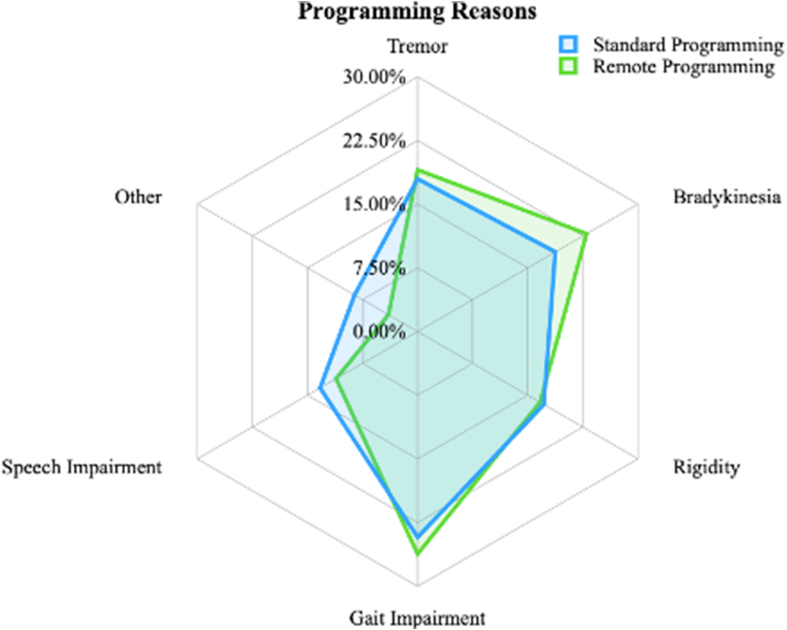
Comparison of programming reasons between two groups. In the radar chart, the blue line represents the frequency distribution of complaints prior to programming in the remote programming group, while the green line represents the corresponding distribution in the standard programming group. Each axis denotes a specific type of complaint, and the values along each axis indicate the percentage of complaints.

Patients in the SP group underwent a total of 44 postoperative programming sessions, averaging 2.2 sessions per person. In comparison, patients in the RP group received 54 sessions, with an average of 2.5 sessions per person. In the RP group, the average duration of the initial programming session was 50 ± 15 min, while subsequent sessions averaged 22 ± 10 min. There was no statistically significant difference in the distribution of subsequent programming reasons between the two groups (Fig. 2, p = 0.686), with gait disturbances being the most reported reason for programming adjustments.

The average stimulation parameters in the SP group were as follows: for the left electrode, 2.66 ± 0.77 V, 52.22 ± 4.24 μs, and 125.19 ± 24.24 Hz; for the right electrode, 2.74 ± 0.77 V, 51.15 ± 6.53 μs, and 134.62 ± 23.06 Hz. In the RP group, the average parameters were: for the left electrode, 2.92 ± 0.67 V, 52.41 ± 9.12 μs, and 130.86 ± 23.45 Hz; for the right electrode, 2.87 ± 0.83 V, 52.14 ± 6.30 μs, and 128.93 ± 25.14 Hz. There were no statistically significant differences between the two groups in the number of programming sessions or in stimulation parameter settings (all p > 0.05).

**Fig. 3:**
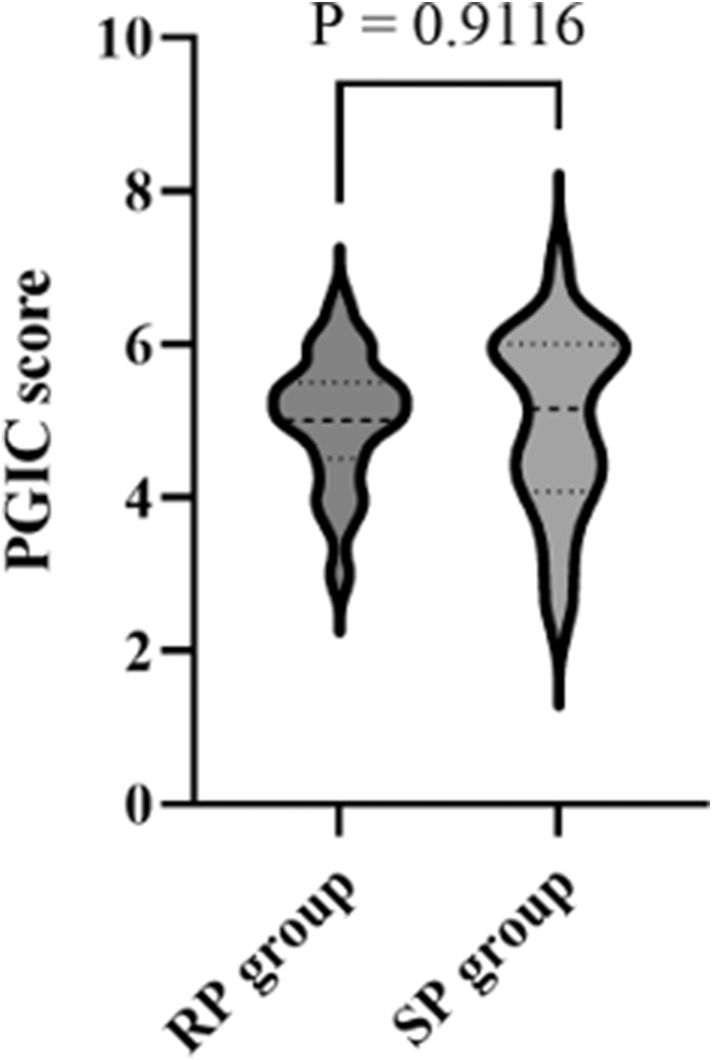
Comparison of PGIC scores after programming in two groups. Abbreviations: PGIC = Patient Global Impression of Change; RP = Remote Programming; SP = Standard Programming.

A total of 93 PGIC scores were collected from patients following programming sessions, including 44 from the SP group (response rate: 100%) and 49 from the RP group (response rate: 91%, 49/54). There was no statistically significant difference in PGIC scores between the SP and RP groups (4.99 ± 1.19 versus 5.03 ± 0.85, p = 0.912; Fig. 3).

During the study, no surgery-related adverse events were observed. Some patients experienced mild stimulation-related side effects following programming. In the SP group, mild dyskinesias and mild tremors were reported 19 and 9 times, respectively. In the RP group, mild dyskinesias and mild tremors occurred 10 and 7 times, respectively. All stimulation-related reactions resolved spontaneously or were alleviated through patient-initiated adjustments to the stimulation intensity. No programming failures due to unstable network connections occurred throughout the RP process.

To investigate whether the programming group influenced patients’ subsequent programming preferences, we analyzed the programming records of all participants from 6 to 18 months following DBS surgery. No significant difference was found between the two groups (p = 0.405; Supplementary Table S3).

## Discussion

The results of this study demonstrate that RP may serve as an effective and safe alternative to the in-person post-DBS management model. In this study, the improvement in motor symptoms among patients in the RP group was non-inferior to that observed in the SP group. Additionally, there were no statistically significant differences between the two groups in terms of subjective improvement following each programming session, nor in the improvement of non-motor symptoms and quality of life. This study employed an open-label design and provides Level III evidence—according to the Oxford Centre for Evidence-Based Medicine.^26^

Although certain motor features, particularly rigidity and postural reflexes, cannot be directly assessed through video-based examinations, DBS programming decisions are not based solely on these components. Other clinically relevant manifestations, including tremor, bradykinesia, gait performance, and treatment-related adverse effects, can be evaluated remotely.^27^, ^28^, ^29^, ^30^ In addition, parameter adjustments were guided by each patient’s previously established therapeutic window and followed by a period of observation to monitor symptom changes and side effects. Therefore, remote programming relies on a combination of video-based assessment and individualized clinical information rather than on the evaluation of any single motor sign. This may explain why motor outcomes achieved with RP were comparable to those obtained with conventional in-person programming.

The benefits of DBS surgery for patients with PD extend beyond improvements in motor symptoms. In this study, both the SP and RP groups showed improvements in mood and quality of life, consistent with findings from previous research.^31^^,^^32^ Additionally, although the difference was not statistically significant, we observed that the medication reduction was less pronounced in the RP group compared to the SP group. This finding aligns with the results reported by Chen et al.,^13^ which may reflect differences in medication adjustment strategies, although this observation should be interpreted cautiously.

In the SP group, three patients requested to switch to RP after surgery due to geographical constraints and were therefore excluded from the study, which reflects a real-world challenge faced by some patients—limited accessibility to postoperative programming services. A previous survey found that many PD patients with DBS reported inadequate access to medical services, with over one-third experiencing difficulty attending in-person visits and over one-quarter having difficulty contacting the clinic for medical advice.^8^ However, this study does not suggest that RP is superior to SP, as no significant differences were observed between the two groups in programming records from 6 to 18 months after DBS surgery, suggesting that maintaining the availability of both programming methods may be more acceptable in clinical practice.

It is worth noting that although both groups were permitted to request additional sessions, RP group underwent a slightly higher average number of total programming sessions (2.5 versus 2.2). This difference raises the possibility that the observed non-inferior outcomes may reflect not only the programming modality itself but also the improved accessibility and increased opportunities for clinical interaction provided by RP. A recent multicenter study reported that enhanced accessibility through RP enabled patients to achieve DBS clinical efficacy (as indicated by a PGIC score improvement of more than one point) more quickly.^33^ In that study, patients receiving RP reached clinical efficacy within an average of 15.1 days post-surgery, compared to 54.2 days in those who only received SP. In contrast, our study did not observe a similar time advantage, as all patients in both groups were scheduled for their first programming session one month after surgery. Future studies should investigate the potential role of programming frequency, rather than modality alone, in influencing motor outcomes.

With the growing adoption of DBS therapy in China, postoperative management has emerged as a significant challenge for both patients and healthcare providers. In this study, the average duration of initial and follow-up programming sessions in the RP group was 50 and 22 min, respectively—both longer than the average consultation time in outpatient clinics at tertiary public hospitals in China (15.3 min),^34^ which highlights a substantial workload associated with SP after DBS surgery. To address this challenge, technological innovations such as closed-loop adaptive stimulation systems and visual programming technologies integrated with imaging data have emerged as active areas of research.^35^^,^^36^ In parallel, connectomic approaches, wearable-based objective monitoring, and AI-assisted programming algorithms are increasingly being explored to enable more precise and individualized DBS management.^37^, ^38^, ^39^ Although these approaches are not yet implemented in clinical practice, RP may provide a suitable platform for the future integration of these evolving technologies.

This study has several limitations. 1. As a single-center clinical investigation, caution is warranted when generalizing the findings to other medical centers, variations in physician experience across centers could affect treatment outcomes. 2. Motor and non-motor outcomes were assessed at a single 6-month follow-up, representing an early phase in the DBS adjustment process. Potential challenges—such as battery depletion, signal instability, and the natural progression of PD—may impact treatment durability and necessitate ongoing programming support.^40^^,^^41^ 3. Although common adverse events such as dyskinesias and tremors were reported, their severity was not systematically evaluated, which may limit the strength of the safety conclusions. 4. The open-label design of this study may introduce performance and detection bias, particularly given the subjective nature of several outcome measures. 5. Due to the absence of recorded data on the duration of traditional outpatient programming sessions, we were unable to quantitatively assess the potential advantages of RP in optimizing medical resource allocation. 6. Secondary outcomes were exploratory and reported without adjustment for multiple comparisons, increasing the risk of Type I error. Future research should aim to comprehensively assess the clinical value of telemedicine by integrating it into broader care workflows, ideally through multicenter, long-term, blinded controlled trials.

To conclude, this study demonstrated that, within six months after DBS surgery, PD patients receiving Internet-based RP achieved motor improvements that were non-inferior to those receiving SP. These findings provide Level III evidence supporting the short-term effectiveness of RP in the postoperative management of this patient population, while studies are needed to evaluate its long-term durability and generalizability in other centers.

## Contributors

X.W.: Participated in the organization and execution of the research project; contributed to writing the draft of the manuscript. Accessed and verified the data.

Y.Z.: Participated in the organization and execution of the research project; contributed to writing the draft of the manuscript.

P.H.: Participated in the execution of the research project and statistical analysis.

Y.P.: Participated in the execution of the research project and statistical analysis.

Z.L.: Conceived the research project and reviewed the manuscript.

Z.Z.: Conceived the research project and reviewed the manuscript.

Y.T.: Reviewed and critiqued the statistical analysis and the manuscript.

C.Z: Reviewed and critiqued the statistical analysis and the manuscript.

P.S: Reviewed the manuscript.

D.L.: Conceived and organized the research project; critically reviewed the manuscript. Accessed and verified the data.

## Data sharing statement

The de-identified individual participant data underlying the results reported in this article will be made available upon reasonable request to the corresponding author.

## Declaration of interests

The authors declare that there are no conflicts of interest relevant to this work.

## Appendix A Supplementary data

Supplementary Tables S1–S3

Study protocol
