# Supplementary material for: Remote programming versus standard in-person programming following deep brain stimulation in patients with Parkinson’s disease: a randomised controlled trial
Source: eClinicalMedicine. 2026 Jul 9;97:104070. doi: 10.1016/j.eclinm.2026.104070 (PMC13380101; doi:10.1016/j.eclinm.2026.104070)
Supplement: Supplementary Tables S1–S3 [file mmc1.pdf]

Supplement Table 1

Initial Programming Record of DBS

Name	Today	Birth year	Surgery date	Disease onset	Note

Contact	mV/ mA	Effect				Side effect				
		Tapping	Rotation	Tremor	Other	Paresthesia	Speech impairment	Facial pulling	Limb spasm	Other
R/3	0									
	1.0									
	2.0									
	3.0									
	4.0									
	5.0									
R/2	0									
	1.0									
	2.0									
	3.0									
	4.0									
	5.0									
R/1	0									
	1.0									
	2.0									
	3.0									
	4.0									
	5.0									
R/0	0									
	1.0									
	2.0									
	3.0									
	4.0									
	5.0									

Contact	mV/ mA	Effect				Side effect				
		Tapping	Rotation	Tremor	Other	Paresthesia	Speech impairment	Facial pulling	Limb spasm	Other
L/3	0									
	1.0									
	2.0									
	3.0									
	4.0									
	5.0									
L/2	0									
	1.0									
	2.0									
	3.0									
	4.0									
	5.0									
L/1	0									
	1.0									
	2.0									
	3.0									
	4.0									
	5.0									
L/0	0									
	1.0									
	2.0									
	3.0									
	4.0									
	5.0									

Parameter and medication adjustment

Supplement Table 2 Comparison of clinical indicators in the SP and RP groups at baseline and follow-up

	Standard programming group			Remote programming group		
	Baseline	Follow-up	p	Baseline	Follow-up	p
Sample size	N = 20			N = 21		
UPDRS-III						
- Total	61.05 ± 11.12	39.00 ± 9.06	<0.001*	63.00 ± 18.08	38.10 ± 10.79	<0.001*
- Bradykinesia	28.25 ± 4.58	20.65 ± 4.88	<0.001*	28.14 ± 7.88	20.43 ± 5.31	<0.001*
- Rigidity	12.75 ± 3.32	8.45 ± 2.74	<0.001*	12.14 ± 3.15	7.57 ± 3.01	<0.001*
- Tremor	10.40 ± 7.00	4.10 ± 3.45	<0.001*	12.29 ± 6.30	4.76 ± 4.21	<0.001*
- Axial symptoms	9.65 ± 3.31	5.8 ± 2.76	<0.001*	10.43 ± 4.28	5.33 ± 2.52	<0.001*
LEDD	855.57 ± 340.86	387.50 ± 179.04	<0.001*	825.59 ± 357.86	427.38 ± 244.17	<0.001*
BDI	10.10 ± 8.13	5.55 ± 3.8	0.005*	9.10 ± 7.62	4.95 ± 4.63	0.027*
BAI	8.95 ± 8.68	3.35 ± 4.87	0.005*	8.24 ± 7.73	4.33 ± 3.38	0.023*
UPDRS-I	9.75 ± 6.29	6.45 ± 3.19	0.014*	8.10 ± 5.55	5.95 ± 3.54	0.010*
UPDRS-II	17.95 ± 8.15	14.3 ± 6.72	0.013*	16.05 ± 6.61	12.24 ± 6.00	0.015*
UPDRS-IV	7.40 ± 2.56	5.10 ± 2.10	0.001*	6.29 ± 2.49	4.95 ± 2.42	0.004*
PDQ-8	8.15 ± 3.86	5.15 ± 3.67	0.006*	7.05 ± 4.95	3.71 ± 2.95	0.001*
EQ-VAS	59.70 ± 18.62	74.20 ± 11.66	0.003*	65.19 ± 16.19	74.62 ± 15.47	0.017*

Abbreviation: LEDD= levodopa equivalent daily dose，BDI= Beck Anxiety Inventory, BAI= Beck Anxiety Inventory, UPDRS= the Unified Parkinson's Disease Rating Scale, PDQ-8=8-item Parkinson’ s Disease Questionnaire, EQ-VAS= EQ-5D Visual Analogue Scale.

*p<0.05.

Between-group comparisons were performed using independent-samples t tests or Mann–Whitney U tests, depending on data distribution.

Supplement Table 3 Programming preferences from 6 to 18 months after DBS surgery in patients initially assigned to the remote programming or standard programming group.

	Standard programming group	Remote programming group	p Value
Programming method			0.405
Remote programming only	6 (24%)	9 (36%)	
Standard programming only	5 (20%)	2 (8%)	
Received both	3 (12%)	6 (24%)	
No programming after last 6 months	11 (44%)	8 (32%)	

Between-group comparisons were performed using the Mantel-Haenszel chi-square test.
