# Supplementary material for: Remote programming versus standard in-person programming following deep brain stimulation in patients with Parkinson’s disease: a randomised controlled trial
Source: eClinicalMedicine. 2026 Jul 9;97:104070. doi: 10.1016/j.eclinm.2026.104070 (PMC13380101; doi:10.1016/j.eclinm.2026.104070)
Supplement: Study protocol [file mmc2.docx]

**STUDY PROTOCOL**

**Protocol title**

Remote Programming for Deep Brain Stimulation in Parkinson’s Disease: A Randomized Controlled Trial

**Short title**

REPRO-PD

**Sponsor and study site**

Sponsor/investigator site: Ruijin Hospital, Shanghai Jiao Tong University School of Medicine, Shanghai, China. The listed study contact is Dianyou Li, MD, PhD.

**Background and rationale**

Deep brain stimulation requires repeated postoperative programming to optimize symptom control. The registered trial is designed to compare remote programming with standard in-person programming in postoperative management of Parkinson’s disease after bilateral subthalamic nucleus DBS. The registry states that the main purpose is to compare improvement in motor symptoms, while also evaluating safety and economic benefit over 6 months.

**Study objectives**

Primary objective

To compare the effect of remote programming versus standard programming on motor improvement after DBS surgery, measured by change in UPDRS Part III from preoperative baseline to 6 months after surgery.

Co-primary objective

To compare economic effectiveness between study groups at 6 months, using quality-adjusted life years estimated from cost data and EQ-5D-3L.

Secondary objectives

To compare between groups:

change in UPDRS Parts I, II, and IV;

quality of life using PDQ-8 and EQ-5D-3L;

mood using BDI-II and BAI;

cognition using MMSE;

patient and caregiver cost burden;

telehealth satisfaction using TeSS;

programming-related adverse events.

**Study design**

This is a randomized, single-center, parallel-assignment interventional study with single masking, enrolling an estimated 50 participants. Participants are randomized before surgery to either a remote programming arm or a standard programming arm. Follow-up continues for 6 months after implantation, with programming visits scheduled at approximately 1, 3, and 6 months postoperatively.

**Study population**

Adults aged 18 to 75 years with primary Parkinson’s disease who meet Chinese diagnostic criteria, are eligible for DBS under the Chinese expert consensus, undergo bilateral STN-DBS, and receive an implanted DBS device with remote programming capability. Participants must have internet access, be able to complete remote assessments, and be trained along with caregivers in use of the remote system.

Inclusion criteria

1. Primary Parkinson’s disease, age 18–75 years.
2. Meets indications for bilateral STN-DBS.
3. Accurate postoperative lead position confirmed by CT.
4. Implanted DBS device supports remote programming.
5. Sufficient home or local internet access for remote management.
6. Participant and caregiver able to communicate effectively and use the remote programming client after training.
7. Able to understand the study and provide consent and comply with follow-up.

Exclusion criteria

1. Preoperative MMSE indicating moderate or worse cognitive impairment.
2. Severe DBS-related postoperative complications such as stroke, encephalitis, or wound infection.
3. Inability to cooperate, understand the study, or provide informed consent.
4. Inability to ensure stable network signal or provide a 4 m × 1.5 m space for movement evaluation.
5. Any other factor judged by investigators to make the participant unsuitable.

**Interventions**

Arm A: Remote programming

After DBS implantation and inpatient training in remote programming, participants receive routine parameter adjustment remotely at 1, 3, and 6 months after surgery. The registry describes remote programming as video-based postoperative management that allows assessment of motor symptoms and parameter adjustment, and may also include device checks, medication adjustment, and psychological counseling.

Arm B: Standard programming

After DBS implantation, participants receive routine in-person programming for device parameter adjustment at 1, 3, and 6 months after surgery. Standard programming is described as conventional post-implant adjustment of IPG parameters based on symptoms during follow-up.
